# A Crosstalk Between Periodontal Disease and Human Immunodeficiency Virus: Application of Artificial Intelligence and Machine Learning in Risk Assessment and Diagnosis—A Narrative Review

**DOI:** 10.3390/dj13120603

**Published:** 2025-12-16

**Authors:** Bhavyasri Gaddam, Leela Subhashini C. Alluri, Ihunna Amugo, Lemlem Berta, McKayla Butler, Shania Ferguson, Alexys Ferguson, Ethel Harris, Vladimir Berthaud, Siddharth Pratap, Qingguo Wang, Chethan Sampath, Zaid H. Khoury, Pandu R. Gangula

**Affiliations:** 1 Department of Oral Diagnostic Sciences & Research, Meharry Medical College, Nashville, TN 37208, USAcsampath@mmc.edu (C.S.); zkhoury@mmc.edu (Z.H.K.); 2 Department of Periodontology, School of Dentistry, Meharry Medical College, Nashville, TN 37208, USA; 3 Department of Internal Medicine, Meharry Community Wellness Center, School of Medicine, Meharry Medical College, Nashville, TN 37208, USA; 4 School of Medicine, Meharry Medical College, Nashville, TN 37208, USA; spratap@mmc.edu; 5 Department of Biochemistry, Cancer Biology, Neuroscience and Pharmacology, School of Medicine, Meharry Medical College, Nashville, TN 37208, USA; qiwang@mmc.edu

**Keywords:** periodontal diseases, human immunodeficiency virus 1, antiretroviral therapy, highly active, artificial intelligence, machine learning, OMICS, precision medicine, biomarkers, narrative review

## Abstract

Periodontal disease (PD) is an inflammatory condition caused by multiple periodontal pathogens, particularly those belonging to the Red Complex. Various risk factors influence the development of PD, including age, sex, socioeconomic status, ethnicity, and underlying health issues. Numerous molecular and cellular processes govern the inflammatory response, which affects the gums and tooth-supporting structures and ultimately leads to alveolar bone loss. Accumulating evidence suggests that Human Immunodeficiency Virus-1 (HIV-1) infection significantly impacts the initiation and progression of PD. While HIV-1 is treated with antiretroviral therapy, this treatment can also affect the course of periodontal disease and systemic health status. AI/ML and precision medicine integrates genomic and computational data to enable individualized disease prevention and treatment strategies. When applied responsibly, these technologies can assist clinicians in the timely detection of both PD and HIV-1. This review aims to discuss the factors that exacerbate PD and the available therapeutic options for persons living with (PLWH) and without HIV-1. Additionally, we emphasize the need for developing biomarkers for early diagnosis and intervention to manage PD effectively, ultimately improving the quality of life for those living with HIV.

## 1. Introduction

### 1.1. Global and Epidemiologic Context

Periodontal disease (PD) is a spectrum of conditions that harm the structures holding teeth in place, known as the periodontium. PD usually starts with gum inflammation (gingivitis) that can heal with proper management but can also progress to irreversible bone damage (periodontitis) and ultimately tooth loss. PD affects approximately 42–50% of U.S. adults and nearly one billion people worldwide with around 750 million individuals experiencing severe forms of the illness. Globally, HIV-1 infection affects 39 million people, including 1.2 million in the United States [1,2]. The coexistence of PD and HIV-1 exacerbates systemic inflammation and accelerates oral and vascular tissue damage. Recent meta-analyses estimate moderate to severe periodontitis in approximately 35–45% of PLWH worldwide [3,4]. The overall prevalence of periodontal and gingival diseases related to HIV-1/AIDS is about 8.2%, making PD a significant oral complication associated with HIV-1/AIDS in both developing and developed countries [5]. In the United States, the prevalence of PD among PLWH is reported to be 62%, with up to 92% of this group also suffering from active dental caries in their permanent teeth [6,7,8,9]. Even with effective viral suppression in HIV-1 patients, oral plaque continues to present a constant microbial challenge [2,10]. Interestingly, the prevalence of PD among PLWH receiving highly active antiretroviral therapy (HAART) has increased, and the reasons for this rise remain unclear [11]. African Americans (AAs) are disproportionately affected by HIV in the U.S. While AAs make up only 13% of the population, they accounted for 42.1% of new HIV-1 infection cases in 2019 [6]. Moreover, AAs living in underserved areas with low socioeconomic status face a greater risk of both PD and HIV infection, along with associated comorbidities such as diabetes, hypertension, and cardiovascular diseases (CVD). This situation is exacerbated by limited access to dental care and ongoing inflammation in oral tissues, which significantly increases the risk of PD among PLWH, particularly within the AA community [12,13]. Although the AA population is notably more vulnerable to PD and HIV-1, as well as associated comorbidities, there is limited knowledge about potential biomarkers that could aid in the early detection of the inflammatory burden causing PD among PLWH [14,15,16]. Furthermore, the relationship between risk factors such as socioeconomic status, diet, and behavior and the systemic spread of pro-inflammatory markers responsible for PD in this population has not been thoroughly explored.

### 1.2. Rationale and Objective

Although individual studies describe immunologic and microbial abnormalities in PLWH with PD, few integrate molecular, therapeutic, and computational dimensions. This review aims to synthesize current knowledge on the bi-directional interaction between PD and HIV-1, emphasizing how multi-omics and AI/ML technologies can enhance diagnosis and risk assessment. The guiding question was: How do immune, microbial, and therapeutic factors interact to influence PD progression in PLWH, and how can AI/ML approaches improve diagnostic precision?

## 2. Materials and Methods

This manuscript follows a narrative review design. A structured literature exploration was conducted in PubMed, Scopus, and Web of Science for publications from January 2010 to May 2025 using combinations of the following keywords and MeSH terms: periodontal disease, HIV-1, antiretroviral therapy, oral microbiome, cytokines, biomarkers, multi-omics, artificial intelligence, machine learning, and predictive diagnostics. Eligible sources included peer-reviewed human, animal, and in-vitro studies, reviews, and meta-analyses published in English. Excluded were conference abstracts without full text, non-English articles, and studies lacking PD or HIV-1 context. Information extracted comprised study design, sample size, PD criteria, HIV-1/ART status, microbiological and immunological findings, and AI/ML methodologies. Because of heterogeneity in study design, a quantitative synthesis was not feasible; therefore, findings were summarized descriptively to highlight mechanistic links and emerging computational applications.

## 3. Etiology of Periodontal Disease

PD is a microbially induced, host-mediated inflammatory disease causing destruction of periodontal ligament and alveolar bone [17]. PD results from microbial dysbiosis that primarily results in a bacterial shift from gram-positive subgingival bacteria to gram-negative bacteria. The orange complex, made up of anaerobic gram-negative species like *Prevotella intermedia*, *Prevotella nigrescens*, *and Prevotella micros*, is the first known microbial complex linked to PD. *Fusobacterium nucleatum,* a member of the Orange Complex, acts as a bridge organism facilitating co-aggregation and biofilm maturation. Contrary to earlier misstatements, these organisms do not “replace” the Orange Complex but increase in relative abundance as pockets deepen, co-existing within polymicrobial biofilms. As the disease progresses, the orange complex changes into the red complex, which is made up of *Tannerella forsythia*, *Treponema denticola*, and *Porphyromonas gingivalis* (*P. gingivalis*) [18]. They coexist with organisms from the Orange Complex, such as *Fusobacterium nucleatum*, *Prevotella intermedia*, *Campylobacter rectus*, and *Eubacterium nodatum*, which serve as ecological “bridges” between early colonizers and late pathogenic consortia. Importantly, the Orange Complex does not transform into the Red Complex; rather, there is a shift in relative abundance as inflammation progresses and local environmental conditions (e.g., oxygen tension, nutrient gradients) change. Within this dysbiotic biofilm, microbial by-products and virulence factors (e.g., lipopolysaccharides, gingipains, and fimbriae) stimulate host immune cells to release proinflammatory cytokines. These host responses drive osteoclast differentiation, connective tissue breakdown, and alveolar bone resorption. Collectively, this community-level pathogenic synergy highlights that periodontal disease results from the interaction between the dysbiotic microbiome and an aberrant host immune response, rather than from infection by individual pathogens.

Risk factors include smoking, systemic diseases (e.g., diabetes, HIV-1 infection), genetic predisposition, and behavioral determinants [19,20]. These can be either modifiable or non-modifiable [21]. These determinants can be grouped into modifiable and non-modifiable categories, as summarized in Table 1. Modifiable risk factors for periodontal disease include both patient-specific and local determinants. Patient-specific factors encompass aging, socio-economic factors, ethnicity and comorbid systemic conditions. Local factors include tooth malposition, high frenal attachment, calculus accumulation and inadequate oral hygiene practices.

### 3.1. Social Determinants

Social determinants are non-medical factors that influence a person’s overall health. These factors include education, employment, family income, physical health, housing, and mental health. Periodontal diseases are particularly prevalent among individuals with lower levels of education, low income, and those of black or mixed ethnicity [22]. Similar patterns of disparity are also observed in HIV, where overlapping socioeconomic and demographic risk factors compound the burden of comorbidities such as periodontal disease. A report by Merchan which focused on adults aged 18 to 64 years found that various social determinants of health, such as socioeconomic status, social security, health insurance, low literacy rates, and dental care utilization, along with behavioral aspects like high tobacco consumption, were significant risk factors for periodontal and other oral diseases [23]. To effectively address social disparities in periodontal health, a shift from an individualized treatment approach to a public health model that targets populations is necessary. Offering toothbrushes and nicotine replacement therapies to patients can help prevent periodontal diseases in socially disadvantaged groups [24].

### 3.2. Sex Bias and Hormonal Influence

Hormonal fluctuations modulate gingival inflammation but are more strongly linked to gingivitis than periodontitis. Elevated estrogen and progesterone levels during pregnancy and menstruation increase vascular permeability and cytokine response to plaque biofilms can be linked to the development of gingivitis [25]. According to Kornman and Loesche, the ratio of bacterial anaerobes to aerobes, as well as the proportions of *Bacteroides melaninogenicus*, *Prevotella intermedia*, and *Porphyromonas gingivalis*, increase during pregnancy [26]. Although some epidemiologic data suggest higher periodontitis prevalence in men, this difference largely reflects behavioral factors (smoking, oral-hygiene practices) rather than endocrine effects. In men, age-related declines in testosterone and vitamin D levels may contribute to bone resorption and impaired repair capacity [27].

### 3.3. Ethnicity Variations

Data from the National Health and Nutrition Examination Surveys (NHANES) I, II, and III indicate that various racial and ethnic groups are disproportionately affected by periodontitis. Specifically, the NHANES data show that Hispanic Americans (HAs) and African Americans (AAs) experience higher rates of periodontitis compared to Caucasian Americans (CAs) [28]. Research by Vlachojanni et al. found that antibodies against *P. gingivalis* were three times more prevalent in AA patients aged 40 and older than in CA patients [29]. Periodontitis is also consistently linked to chronic systemic conditions such as cardiovascular disease, cancer, and diabetes, which themselves exhibit racial and ethnic disparities [29,30]. These systemic health conditions are often worsened by social determinants of health, including limited access to care, poverty, rural living, unemployment, inadequate housing, and food insecurity, as well as structural racism and discrimination.

### 3.4. Classification of Periodontal Diseases

The 2018 AAP/EFP classification categorizes periodontal diseases by stage (severity and extent of tissue loss) and grade (rate of progression and risk factors). This replaces the older chronic/aggressive taxonomy. This classification of periodontal and peri-implant diseases and conditions is based on the severity, complexity and extent of a patient’s periodontal disease [30,31]. One of the categories of periodontitis include: Necrotizing periodontal diseases such as necrotizing gingivitis (NG), necrotizing periodontitis (NP) and necrotizing stomatitis (NS) (which are characterized by papillary necrosis/punched-out interdental papilla, bleeding, extreme pain, and an impaired immune response) and linear gingival erythema, can be observed in HIV-1 patients [32].

## 4. Human Immunodeficiency Virus Infection (HIV)

Human immunodeficiency virus infection is a global health epidemic and if left untreated, results in fatal consequences. Several systemic health complications such as diabetes, cardiovascular disease, gastrointestinal disease, and cancer have been documented among PLWH [32,33]. However, PD is the most common oral health problem noticed among persons living with HIV [PLWH] [34]. A projected 1.3 million individuals contracted HIV in 2023 and 630,000 people died from HIV related causes. By 2025, 95% of all HIV positive individuals should have received a diagnosis, while 95% should be receiving life-saving antiretroviral therapy, and 95% of those receiving treatment should have their viral load suppressed for both personal health reasons and to prevent HIV from spreading to others [34]. These figures were 86%, 89%, and 93% in 2023, respectively. Of all HIV-1 positive individuals in 2023, 86% were aware of their status, 77% were on antiretroviral medication, and 72% had suppressed viral levels [4].

### 4.1. Etiology of HIV Infection

HIV is a member of the Lentivirus genus and family, Retroviridae. HIV is classified into two basic types: type 1 (HIV-1) and type 2 (HIV-2). The HIV-1 and HIV-2 genomes differ significantly in terms of amino acid composition despite sharing a similar structure. The two viruses differ significantly in terms of severity, transmissibility, and prognosis because they are the products of two distinct zoonotic transmissions of simian immunodeficiency viruses. Compared to HIV-1, HIV-2 is less commonly acquired, mainly prevalent in West African countries, and is associated with a less severe disease course. It should be noted that HIV-1 and HIV-2 only share 48% identity at the nucleotide level and only 60% identity at the amino acid level [35]. There are various modifiable and non-modifiable risk factors associated with HIV and care, also associated with periodontitis (Table 2). The primary target of the virus is CD4+ T-lymphocyte helper cells, which results in a severe type of immunological subversion and a persistent loss of these cells. This impairs the immune system and results in numerous clinical symptoms of the disease. When HIV infection is left untreated, it might eventually lead to acquired immunodeficiency syndrome (AIDS). The immune system cannot fight off infections at this point, which leads to opportunistic infections and death [4,35].

Few of the risk factors such as race ethnicity, systemic conditions such as metabolic syndrome, obesity are common for both HIV-1 and periodontitis. Various body fluids such as blood, amniotic fluid, breast milk, semen, pre-ejaculate, rectal fluids, and vaginal fluids can transmit HIV-1. Sexual intercourse, vertical transmission i.e., from mother to child, fomites such as injection drugs or reusable medical equipment HIV-1 can be some of the major forms for HIV-1 transmission [34].

### 4.2. Management of HIV

Individuals who encounter HIV-1 positive infectious body fluids by direct mucous membrane contact, skin punctures, or skin injury are susceptible to HIV-1 transmission. The probability of HIV-1 infection following exposure depends on the density of CD4-positive cells and the number of virions at the exposure site. Antiretroviral treatment ought to begin as soon as exposure to HIV-1 infected fluids occurs. US Public Health Service guidelines, post-exposure prophylaxis (PEP) should be started up to 72 h after exposure [36]. Combination ART medication with emtricitabine + tenofovir plus raltegravir for four weeks is the suggested regimen [37]. Follow-up HIV testing should be performed on those who have been exposed to HIV-1 at 6, 12, and 24 weeks. At 24 weeks, if the test results remain negative, the individual is deemed to be non-infectious. Tenofovir alafenamide/emtricitabine was authorized by the FDA in 2019 as a pre-exposure prophylactic for adults and adolescents weighing at least 77 pounds (35 kg). Current FDA-approved antiretroviral drugs are listed in Table 3, categorized by their mechanisms of action. Antibodies towards HIV-1 develop between 6 weeks to 6 months post initial infection. HIV-1 treatment plans are frequently modified in response to side effects and possible drug combinations with the patient’s existing prescriptions. Patients who are expecting should start treatment right once to safeguard the woman’s health and stop HIV-1 from being passed from mother to child [38].

### 4.3. Administration of Medications in HIV-1

Preventing HIV-1 mutation is one of the important indications to provide standard of care in an HIV-1 regime. Since these prescriptions must be taken orally by the patient, there are now several choices that combine three to four medications into a single pill, which should improve patient compliance because they only need to be taken once daily. This dosage improves long-term efficacy as well as adherence. Because Ibalizumab is an injectable medication, it is an exception [35,46].

#### HAART Therapy

Highly active antiretroviral therapy (HAART) also called as antiretroviral therapy or combination antiretroviral therapy is a treatment regimen typically consisting of a combination of three or more antiretroviral drugs [47]. The primary goal of HAART therapy is to reduce the transmission of HIV-1 to others. Different drugs which inhibit viral replication are incorporated into a single drug which is the key cornerstone of HAART therapy. This approach can help in optimizing patient care and help in improving patient outcomes [48].

*Goals of HAART in Patients with HIV-1 Infections*:

- Improve quality of life (QOL)
- Reduce plasma viral RNA load.
- In Acquired immune deficiency syndrome and non-AIDS cases, reduce morbidity and mortality.
- Prevent transmission to others such as needle sharing partners, sex partners and mother to infant.
- Improve immune function and
- Prevent drug resistance

### 4.4. Evaluation

HIV-1 viral load should be checked every four to eight weeks after starting HAART until the levels drop below the assay’s limit of detection. Despite patient medication adherence, if viral suppression does not occur after 24 weeks, healthcare practitioners should be tested for resistance and change the patient’s prescription. Following HAART regimen stabilization, follow-up appointments are to be planned for every three to six months. To monitor progress, it is recommended that all patients have their HIV-1 RNA viral load and CD4 count checked. Additionally, repeats of the complete blood count with differential, blood urea nitrogen and creatinine, fasting glucose/hemoglobin A1C, alanine and aspartate aminotransferases, and total bilirubin should be performed to screen for medication toxicity. The BMI and waist may also be measured at follow-up visits [48].

OraQuick is a rapid point-of-care (POC) HIV-1 test that has been developed recently which can provide results in 20 min. This helps to speed screening and correctly detect HIV infection in periodontal patients. The fluid to be diagnosed is combined with a developing solution in a vial, and a testing instrument shows the outcomes. It is a stick-shaped instrument that is placed into a tube of testing fluid and has a cloth swab on one end. The first oral swab in-home test for HIV-1 and HIV-2 authorized by the FDA is called OraQuick^®^ [39].

### 4.5. HIV-1 and Periodontal Disease

**Figure 1:**
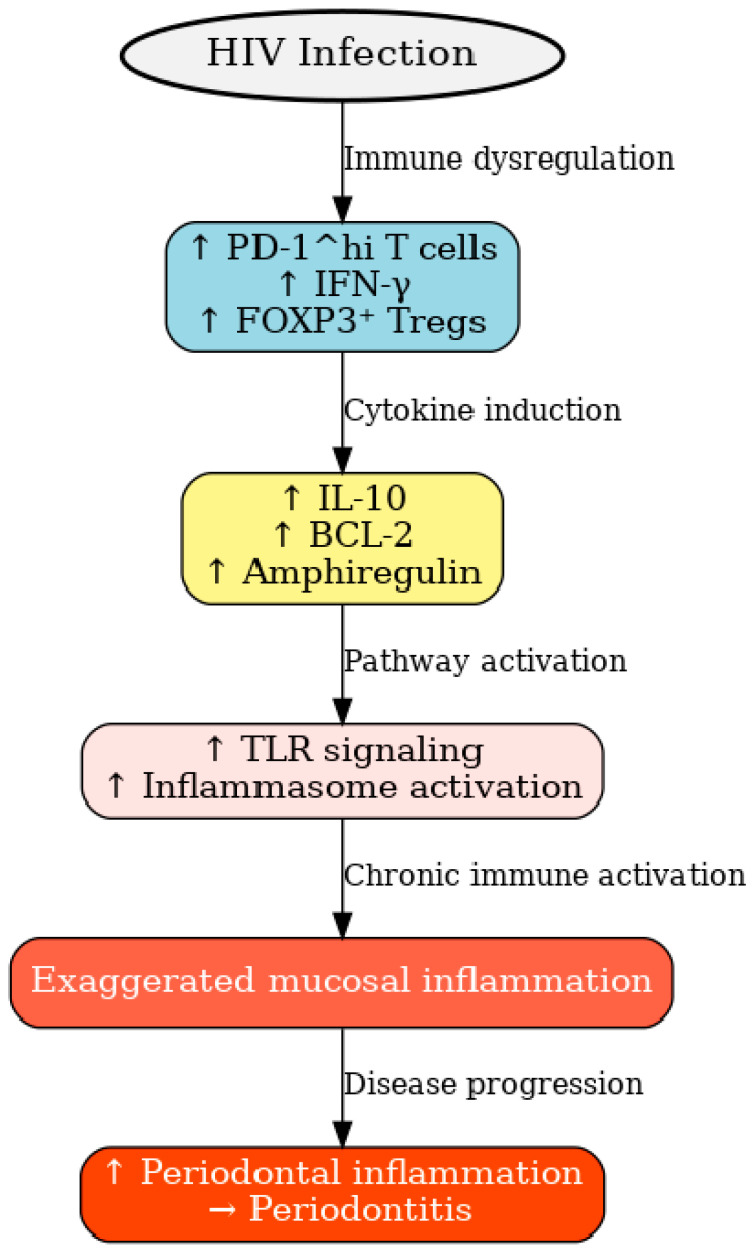
Factors associated with periodontitis in HIV patients. HIV infection induces immune dysregulation characterized by increased PD-1^hi T cells, IFN-γ, and FOXP3^+^ Tregs. These changes elevate IL-10, BCL-2, and amphiregulin, which enhance Toll-like receptor signaling and inflammasome activation. The resulting chronic immune activation drives exaggerated mucosal inflammation, leading to periodontal tissue damage and progression of periodontitis. Up arrow indicates higher, leading to periodontitis.

HIV-1 is always associated with immune deficiency and can cause other inflammatory diseases to initiate or aggravate. Elevated infiltration of Programmed cell death protein-1 high (PD-1^hi) interferon-gamma positive (IFN-γ^+^) forkhead box P3 positive (FOXP3^+^) T cells has been observed in chronic inflammatory conditions such as periodontitis and in HIV-infected tissues, reflecting a state of immune exhaustion and regulatory dysfunction. PLWH exhibit an increased population of PD-1^hi IFN-γ^+^ FOXP3^+^ regulatory T cells, which are associated with elevated expression of interleukin-10 (IL-10), B-cell lymphoma 2 (BCL-2), and amphiregulin, contributing to an immunosuppressive microenvironment compared to healthy individuals. It also upregulates toll-like receptors and inflammasome pathways with CD4+ cells which show hyperactivated phenotype (Figure 1). This process subsequently increases periodontal inflammation [38].

### 4.6. How Is Antiretroviral Treatment Associated with Periodontal Inflammation and HIV-1?

Anti-retroviral treatment also has a few adverse effects, which include increased markers of premature aging, bone mineral loss, oral and intestinal dysbiosis, and persistent low-grade inflammation. However, with HAART, it seems that the landscape of HIV-1-related illnesses has shifted, with the virus now being connected to the worsening of aging and chronically inflammatory-related conditions [49]. According to Glass et al., periodontitis may contribute to poorer HIV-1 management because it simultaneously triggers immunological activation brought on by HIV-1 and chronic periodontitis, exacerbating the systemic inflammatory state and jeopardizing treatment. Furthermore, inflammatory gingival tissue can serve as a reservoir for HIV-1, which can aid in the virus’s reactivation and pose a challenge to the elimination and management of HIV-1 [50].

Additionally, there is a close connection between systemic disorders and periodontitis, particularly in terms of immunological and inflammatory responses. Diabetes, heart disease, rheumatoid arthritis, and metabolic syndromes are a few of these ailments. This is a crucial aspect to consider because several chronic inflammatory diseases can interact with one another and amplify the pathological consequences of the afflicted illnesses [51,52,53].

Early periodontitis can increase salivary and serum concentrations of NLRP3, an important inflammatory marker that governs the activation of IL-1β and its pro-inflammatory effects, which include the recruitment of neutrophils and other cells innate immune systems [54]. This is supported by recent studies. Increased levels of this biomarker have also been linked to complications in the management of long-term conditions such as diabetes, heart disease, and HIV-1 [55].

Analyzing the potentially harmful effects of the virus in influencing chronic periodontitis requires ongoing research into the risk factors and prevalence of periodontitis in people with and without HIV-1 [56,57,58]. Given that periodontitis can interact with the HIV-1 virus and make clinical care of the illness challenging, it is a condition that needs to be avoided and managed, particularly in those who are HIV-1 positive. Furthermore, the detection and validation of potential risk factors associated with periodontitis facilitates the development of public and commercial health initiatives targeted at mitigating the disease’s effects on susceptible groups [59]. Because the infection itself contributes to the deterioration of the oral mucosal epithelium, it favors microbial translocation and induces a systemic inflammatory state, which in turn influences the prevalence and severity of periodontitis in this population [10]. Systemic immune activation and potential amplification of periodontal inflammation come from a reduced synthesis of interleukin-17 (Th17) and interleukin-22 (Th22) cells in these cells due to high virus replication and substantial depletion of CD4+ T lymphocytes [60].

HIV-1-infected adults with low CD4+ T-cell counts have been demonstrated to have twice the risk of clinical attachment loss and tissue degradation compared to non-infected controls. The assessment of CD4+ T lymphocyte count is a crucial technique for tracking the progression of HIV-1. Its depletion in number and altered function results in immune response suppression, an increase in oral opportunistic infections, and other periodontal disorders [56]. Atypical periodontal diseases appear to be more likely to occur in people who do not receive antiretroviral medication because of the significant decrease in immunity. Furthermore, the cytokine network and the number of macrophages, leukocytes, and dendritic cells are regulated by the rise in T-cell count brought about by HAART, which increases the organism’s ability to fight infections [54,55].

### 4.7. Molecular and Cellular Events in PD With or Without HIV-1 Infection (With or Without ART) Compared to Healthy State

Periodontal disease is characterized by complex and dynamic interplay between host cellular and molecular levels. A healthy periodontium maintains a balance between pro-inflammatory and anti-inflammatory mediators. When this balance is disrupted, and pro-inflammatory mediators increase, periodontal inflammation, and destruction occur [61]. Once periodontal inflammation begins, various pro-inflammatory mediators, such as neutrophils, macrophages, and eosinophils, are released. These mediators also promote the simultaneous release of anti-inflammatory mediators to help balance the inflammatory factors during periodontal inflammation. Inflammatory immune reactions are triggered when tissues are damaged by microbial invasion and through antigen presentation, which involves antigen-presenting cells like dendritic cells [49].

Additionally, dendritic cell receptors are sometimes targeted to enhance antibacterial responses efficiently. Myeloid dendritic cells (DCs) are mainly targeted to improve these immune responses and activate T-cells [50]. This interaction alters the behavior of endothelial and parenchymal cells and the periodontal pathogens associated with lipopolysaccharides (LPS) and other immune cells. Gram-negative bacteria contain LPS in their cell walls, prompting a reactive response from the host [62]. Among the proteins that regulate apoptosis, inflammation, and immunity are toll-like receptors (TLRs) [15,16]. *P. gingivalis* has various virulence factors which help in progression of periodontal disease. These include fimbriae (long and short), hemolysin, hemagglutinins, capsule, outer membrane vesicles (OMVs), lipopolysaccharides (LPS), and gingipains. By producing IL-1β, TNF-α, and IL-6, small fimbriae in *P. gingivalis* aid in the differentiation of osteoclast precursor cells into osteoclasts and promote bone resorption. By interfering with (down-regulating) the production of chemokines (referred to as local chemokine paralysis) and cell adhesion molecules like IL-8, ICAM-1, and E-selectin that are essential for leukocyte diapedesis, *P. gingivalis* might hinder the recruitment of neutrophils [63].

*P. gingivalis* produces high local concentrations of C5a ligand through the expression of ligands that activate the Toll-like receptor 2 (TLR1)–TLR2 complex and enzymes (HRgpA and RgpB gingipains) with C5 convertase-like activity. By co-activating C5aR and TLR2 in neutrophils, the organism can prevent a host-protective antimicrobial response by causing ubiquitination and proteasomal degradation of the TLR2 adaptor MYD88 [63]. SerB phosphatase has a variety of roles in host cell interactions with *P. gingivalis*. The NF-kB transcription factor, which controls the synthesis of IL-8, is strongly and selectively inhibited by this enzyme, which is released by *P. gingivalis*. Similarly, *T forsythia* has a typical chemotactic ability and can sense host stimuli while significantly affecting inflammatory lesions and periodontal disease progression [64,65,66]. The Hydrolytic, proteolytic, and lipolytic proteases in *P. gingivalis* cause periodontal breakdown [63]. It also co-aggregates with other bacteria in the formation of biofilm and acts as a bridge between early and late colonizing bacteria [67,68,69]. Certain TLRs are expressed in the periodontal inflammatory response in clinically healthy gingiva, contrasting with the previously mentioned processes [4]. Activated dendritic cells can amplify the initial activation signal received by T cells from their receptors, facilitating the development of specific cytokine patterns [70]. These cytokine patterns are crucial for developing CD4 T-cell life cycles and specific migratory patterns. When host cells encounter microbial dental biofilm, pro-inflammatory cytokines such as tumor necrosis factor and interleukins are released [50,62]. Other products released during the acute phase of inflammation include arachidonic acid (AA) and complementary factors like thromboxane and prostacyclin. Research has shown that the most effective cytokines for inducing bone resorption through RANK ligand activation, thereby enhancing osteoclast activity, are IL-6 and IL-1β [4,62]. IL-1 has three types: IL-1β, IL-1α, and IL-1. The catabolic activity of these three cytokines is regulated by endogenous inhibitors known as IL-1 and TNF receptor antagonists. These antagonists can help reduce infection and decrease the severity of inflammation [70].

### 4.8. Genomic Medicine in Periodontal Disease and HIV-1

Genomic medicine is another field of medicine that focuses on using the genetic information of a person for management, individualized treatment, diagnostic, and therapeutic tailoring. Genomic medicine is now combined with precision medicine to improve conventional symptom-driven medicine to multi-omics profiles where the management of disease is based on demographic, epidemiological, clinical, and imaging data. Predictive, preventive, personalization, and participatory are 4 p’s which have been focused on by these genomic and precision medicine. Its potential has already been shown in fields of pharmacology, oncology, infectious diseases, and inflammatory conditions [71]. Precision medicine aims to assist physicians in swiftly understanding how individual clinical data differentiation can impact the diagnosis of health conditions and diseases and predict the optimal dosage of treatment for each patient. Several lines of evidence suggest that biotechnology is developing rapidly and when is utilized in medicine, artificial intelligence has been discovered which helps to uncover many technical advancements in various fields of medicine [72]. In periodontology, host genetic susceptibility has been linked to dysregulated inflammatory pathways, alveolar bone resorption, and differential responses to microbial colonization. Variants in cytokine genes (e.g., IL-1, IL-6, TNF-α) and immune-regulatory loci have been associated with increased risk of severe periodontitis, suggesting that genomic profiles could stratify patients by disease susceptibility and progression. In HIV-1 infection, genomic factors such as HLA alleles, CCR5 polymorphisms, and cytokine gene variants influence immune function, viral load dynamics, and susceptibility to opportunistic oral infections. Integrating genomic medicine into oral health research provides opportunities to identify predictive markers that link host immune genetics with microbial dysbiosis and systemic inflammation, advancing toward individualized prevention and therapeutic strategies for HIV-associated periodontitis.

## 5. Diagnostic and Biomarker-Based Approaches in Periodontal Disease and HIV

### 5.1. Conventional Clinical Assessment

Diagnosis of periodontal disease is important in understanding its etiology and suggesting appropriate evidence-based treatment to patients. Rapid diagnosis can help in a rapid treatment plan and rapid recovery. The gold standard for diagnosis involves a thorough clinical periodontal examination such as probing-pocket depth (PPD), clinical-attachment loss (CAL), bleeding on probing (BOP), supported by intraoral radiographs [73,74]. A few novel techniques have been discovered to diagnose periodontitis based on CRP levels, biomarkers, etc. The following are some of those.

### 5.2. Emerging Point-of-Care Diagnostics

Diagnosing periodontal disease is crucial for understanding its causes and recommending effective evidence-based treatments for patients [39,75]. A rapid diagnosis allows for developing a quick treatment plan, leading to faster recovery. Several innovative techniques have been developed to diagnose periodontitis based on CRP (C-reactive protein) levels and other biomarkers. Here are some of these novel methods:

#### 5.2.1. Electronic Taste Chips

Researchers at Rice University in Houston, Texas, have created a lab-on-a-chip system called the Electronic Taste Chip (ETC). This device uses CRP levels to differentiate between individuals with periodontal disease and those who are healthy. Using a microchip-based detection system, the ETC measures analytes (such as acids, bases, electrolytes, and proteins) in a solution. Chemical and immunological interactions occur on a sensor array positioned within microspheres located in inverted pyramidal microchambers on the microchip [75]. Charge-coupled devices generate varied optical signals from these reactions, which are captured and recorded by a CCD (Charge-Coupled Device) video chip. The porous beads in the ETC provide a significant advantage over traditional ELISA methods, as they allow more antibody molecules to bind and detect CRP even at low concentrations. In contrast, antigen-antibody interactions in ELISA occur on a single layer at the bottom of the well [76].

#### 5.2.2. Integrated Microfluidic Platform for Oral Diagnostics (IMPOD)

This point-of-care diagnostic test allows for rapidly quantifying salivary biomarkers associated with oral diseases. Combining sample pretreatment with electrophoretic immunoassays, the IMPOD facilitates the quick determination of analyte concentrations in minimally pretreated saliva samples. This technology enables hands-free analysis of saliva, allowing for the swift assessment of collagen-cleaving enzyme MMP-8 levels in healthy individuals and those with periodontal disease. Only small volumes of saliva (10 µL) are required, and measurements of MMP-8 and other biomarker concentrations can be completed in 3 to 10 min using this handheld device [77].

These innovations represent significant advancements in the quick and accurate diagnosis of periodontal disease and are seen and recorded by a Charge-Coupled Device (CCD) video chip. The porous beads in the ETC system give it an edge over the ELISA because they enable a higher number of antibody molecules to bind to and detect CRP at very low concentrations. Antigen-antibody interactions in ELISA occur on a single layer at the well’s bottom [33].

### 5.3. Molecular Biomarkers

#### 5.3.1. Matrix Metalloproteinases (MMPs)

MMPs play a crucial role in regulating both physiological and pathological processes. They aid in reshaping cytokines, repairing damaged tissues, remodeling the extracellular matrix, and activating defensins [78]. Additionally, MMPs mediate and regulate immunomodulatory responses by producing cytokines that modify non-matrix substrates, such as chemokines, thereby enhancing healing processes and prioritizing growth factors for cells and tissues. MMPs are also relevant in various pathological conditions, including tissue destruction, periodontal disease defense mechanisms, and wound healing. MMP-2, MMP-9, and MMP-8 are mainly involved in these processes [51,79].

#### 5.3.2. RANK/RANKL/OPG Interactions

Bone resorptions in the development of periodontal disease are mainly influenced by RANK, RANKL, and OPG systems. The interactions between RANK/ RANKL or OPG can lead to bone resorption during the periodontal inflammatory process. Osteoclasts are the cells that consist of RANK, the activation receptor of NF-kB. Its ligand, RANKL, a ligand of RANK is a transmembrane protein expressed on cells such as osteoblasts and activated T cells [78]. During the bone resorption process in periodontitis, RANKL interacts with precursors of osteoclasts as well as cementoclasts, and binds to its RANK receptor to induce bone resorption. Another factor known as Osteoprotegerin (OPG) is a TNF family member expressed in cementoblasts, osteoblasts, fibroblasts, and T lymphocytes. This factor helps inhibit bone resorption by binding to its RANKL. This, in turn, prevents RANK from binding to ligand.

In inflammatory states of the tissue, T cells are activated and produce RANKL. This leads to the modulation of osteoclastogenesis and bone resorption, ultimately resulting in bone loss in periodontitis. In contrast to gingivitis, advanced periodontal disease had higher levels of RANKL expression. This shows that RANKL is involved in the breakdown of periodontal tissues and that inhibiting it may reduce the rate at which periodontal bone is reabsorbed [70]. Anti-retroviral medications can help reduce the oral manifestations caused by viruses. These manifestations include Kaposi’s sarcoma, oral candidiasis, and necrotizing periodontal disease [79].

### 5.4. Multi-Omics Biomarker Discovery

Interactive, user-friendly, multi-omics platforms that merge genomics, transcriptomics, proteomics, and metabolomics have allowed non-computational researchers to better access advanced data analyses. IntelliGenes combines gradient-boosted decision trees with a drag-and-drop interface, supporting nested cross-validation; pilot data already establish gene-metabolite signature that distinguish mild from severe bone loss with over 90% accuracy [80]. Metagenomics covers a complete set of microbial communities inhabiting the oral cavity, revealing shifts in bacterial diversity and enrichment of keystone pathogens (e.g., *P. gingivalis*, *T. denticola*, *T. forsythia*) as well as opportunistic taxa (*Prevotella*, *Fusobacterium*, *Veillonella*) in people living with HIV. Shotgun metagenomic sequencing enables pathway-level predictions, linking microbial gene content to virulence, metabolic potential, and immune modulation. Huang et al. showed that using 16S rRNA metagenomic approach identified six genera, *Filifactor*, *Porphyromonas*, *Treponema*, *Tannerella*, *Aggregatibacter*, and *Peptostreptococcus*, were found to be significantly enriched in the subgingival plaque samples in severe periodontitis compared to healthy controls [81]. Shotgun sequencing has also enabled the discovery of novel taxa, such as *Candidatus* Bacteroides *periocalifornicus*, which shows strong associations with the red-complex bacteria and may represent an emerging periodontal pathogen. These results indicate that microbial transition might play a crucial role in periodontitis pathogenicity.

While metagenomics reveals the taxonomic composition of the periodontal microbiome, metatranscriptomics provides a real-time snapshot of functional microbial activity. Studies demonstrate consistent upregulation of genes controlling butyrate production, iron acquisition, lipopolysaccharide synthesis, and flagellar assembly, promote exacerbate periodontal inflammation and tissue destruction. Moreover, transcriptional differences between stable and progressing lesions, including shifts in cobalamin biosynthesis, proteolysis, and potassium transport, suggest that metatranscriptomic signatures can serve as biomarkers of disease progression. This layer of omics is valuable in distinguishing between merely “present” versus “functionally active” organisms in PD/HIV-associated dysbiosis. These insights emphasize that the pathogenic potential of oral biofilms lies not only in microbial presence but in their active expression programs, a concept especially relevant in HIV-associated periodontitis, where systemic immune dysfunction may amplify the impact of these functional microbial outputs.

### 5.5. Proteomic and Metabolomic Insights

Proteomics and metabolomics provide complementary layers that directly connect microbial activity to host responses. Proteomic profiling of saliva and gingival crevicular fluid has identified alterations in cytokine, chemokine, and matrix-degrading enzyme networks in periodontitis, many of which are further accentuated in PLWH. Elevated expression of inflammatory mediators, host proteases, and complement proteins has been linked with disease severity and tissue destruction. In parallel, metabolomic analyses reveal how small-molecule metabolites serve as mediators of oral–systemic crosstalk. Short-chain fatty acids, amino acid derivatives, and lipid mediators produced by dysbiotic oral biofilms can drive inflammatory cascades, influence bone resorption, and modulate vascular and immune functions. In HIV infection, these metabolites may act synergistically with systemic immune dysregulation, amplifying chronic inflammation and increasing the risk of systemic comorbidities. Together, proteomic and metabolomic studies bridge microbial function with host physiology, providing candidate biomarkers and therapeutic targets that extend the multi-omics framework beyond microbial profiles to integrated host–microbe interactions.

Algorithmic research is nascent regarding PLWH. The National Institutes of Health (NIH) currently funds a multi-center project that couple’s salivary microbiome shifts, longitudinal CD4/CD8 trajectories, and antiretroviral pharmacokinetics in federated machine-learning models designed to predict necrotizing periodontal episodes; early clustering analyses suggest distinct inflammatory ecotypes within virally suppressed cohorts [82]. State guidelines have begun the implementation of this work and have recommended AI-assisted risk stratification when scheduling periodontal recall for immunocompromised adults [82].

## 6. Artificial Intelligence/Machine Learning in Periodontal Disease & HIV

**Figure 2:**
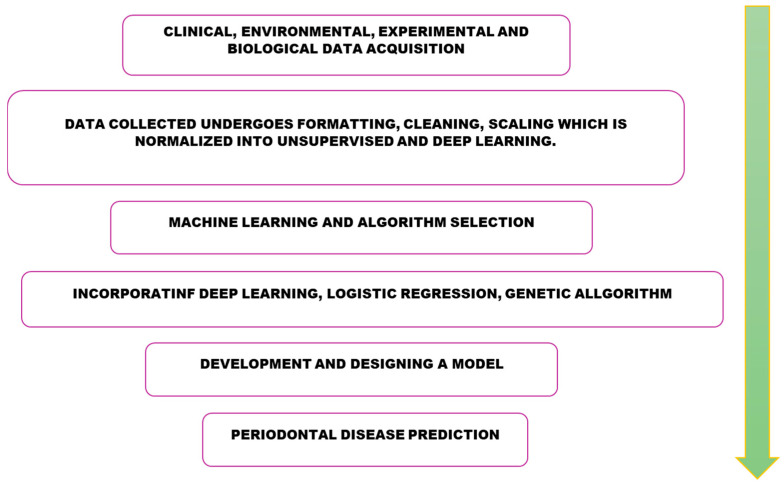
Role of artificial intelligence and machine learning in prediction of periodontal disease. Acquisition from clinical, environmental, experimental and biological data will undergo formatting, cleaning, scaling where it is unsupervised and subjected to machine learning and algorithm selection. This data is incorporated with deep learning, logistic regression and genetic algorithms. These algorithms are utilized to develop and design a model for periodontal disease prediction.

Artificial intelligence (AI), machine learning (ML), and deep learning (DL) have rapidly expanded as key tools in biomedical research, enabling integration of heterogeneous data sources such as electronic health records (EHRs), imaging, and multi-omics datasets. In dentistry and oral medicine, AI/ML is already being used to automate data retrieval, summarize clinical notes, and predict disease outcomes. Neural networks and other ML models have demonstrated value in integrating metagenomic, proteomic, and clinical data to identify microbial–host signatures that distinguish health from disease. For periodontal disease, AI-driven classifiers can detect microbial dysbiosis, predict progression to severe alveolar bone loss, and stratify patients by inflammatory biomarker patterns. In HIV-1–associated periodontitis, explainable AI models could integrate clinical, immunological, and genomic data (e.g., CD4 counts, viral load, cytokine signatures) to improve risk assessment and tailor interventions. Cloud-scale data storage and federated learning approaches may further enable multi-site, multi-ethnic model training, addressing health disparities in populations disproportionately affected by HIV and periodontal disease. Machine learning and deep learning have gained popularity in developing critical components of biomedical data analysis. In medicine, artificial neural networks, machine learning, and deep learning have been categorized into a tremendously emerging branch called artificial intelligence. Currently, this is being used to merge health records, automate data retrieval from sources, summarize EHRs or handwritten physician notes, and store data on a cloud scale [59]. The steps of the using AI/Ml in predicting periodontal disease has been represented in the Figure 2.

Precision medicine is a fast-expanding field of medicine and is the most exciting as well as promising advancements in modern medicine which is focused on the genetic composition, way of life, expression of genes, and surrounding environment as well as the environment of the human body [55]. This field provides efficient expenditure and better patient results by transforming healthcare from a mass medical practice to individualized tailored medicine. This has been applicable in diseases such as cancer, HIV, cardiovascular disease, other inflammatory conditions and more recently, periodontal disease [13,83].

### 6.1. Artificial Intelligence in Periodontal Disease and HIV

PD is a complex disease that poses a challenge for the clinician to diagnose and manage. There are many ways to diagnose PD, but accurate diagnosis remains a challenge. This challenge can be overcome by artificial intelligence and machine learning. The intersection of HIV immunopathology and periodontal inflammation presents a unique data environment characterized by longitudinal immune markers, HAART pharmacodynamics, and distinct microbiota. Pilot studies integrating these layers through ensemble learning have collected salivary biomarker and longitudinal data to train federated ensemble models aimed at predicting necrotizing periodontal episodes in virally suppressed adults [84]. Translating such models to practice requires pipelines that keep patient-level data within HIV clinics while exchanging parameter updates, thereby satisfying both HIPAA and NIH data-sharing mandates.

Explainable AI techniques ought to accompany these efforts; counterfactual analysis can quantify the marginal risk increase conferred by a 10-unit drop in CD4 count at a constant probing-depth trajectory, offering clinicians an intuitive narrative for shared decision-making. Alignment with the New York State Department of Health AIDS Institute (NYSDOH) clinical framework ensures that AI outputs map onto established treatment pathways, facilitating regulatory acceptance and reimbursement [85]. Continued cross-disciplinary collaboration among periodontists, infectious-disease specialists, data scientists, and ethicists is essential to translate these technical advancements into equitable oral-systemic health outcomes.

Ferrara et al. identify several challenges in the widespread adoption of AI in academic and clinical periodontal practice [86]. Challenges discussed within their review include data quality and standardization, interpretability and explainability of AI models, clinical validation and generalizability, integration with existing workflows, ethical and legal considerations, cost and accessibility, and clinician training and acceptance [87]. In terms of data quality and standardization, AI model performance depends heavily on the quality and consistency of training data. Further, there are significant hurdles in standardizing data collection protocols and ensuring high-quality, diverse datasets across different clinical settings. Many deep learning models operate as ‘black boxes’, making it difficult for clinicians to understand and trust their decision-making processes which is an issue of interpretability and explainability [87]. Developing interpretable AI models is crucial for clinical acceptance and regulatory approval. While AI models often perform well in controlled research settings, their performance in diverse real-world clinical environments needs further validation. Ensuring models generalize across different patient populations and clinical settings is a major challenge. Integration with existing workflows needs to be researched more extensively as well. Seamlessly incorporating AI tools into existing clinical workflows without disrupting established practices or increasing clinician workload remains a significant challenge. In summary of these challenges is the shortcomings faced by cost and accessibility, and clinician training and acceptance. The implementation of AI technologies may require significant financial investment, potentially limiting accessibility, especially in resource-constrained settings. Ensuring that dental professionals are adequately trained to use and interpret AI tools, and fostering acceptance of these technologies among clinicians, presents ongoing challenges.

### 6.2. Predictive Analytics from Routine Chair-Side Data

Recent work has shifted the discussion of precision periodontology from theoretical potential to clinically validated algorithms. Machine-learning models trained on multidimensional clinical variables using gradient-boosting and deep-neural ensembles, can now predict treatment response with an area under the precision-recall curve (AUPRC) of 0.90. These results demonstrate that feature-rich yet routinely collected, chair-side data is sufficient for actionable prognostication [88]. This study recorded pocket reduction in a period of 3 months. If implemented clinically, this machine learning approach could potentially change current periodontal treatment practices in significant ways [89].

The described model presents potentiality whereby clinicians can tailor treatment plans to individual patients based on their specific demographic, clinical, and microbiological profiles. This would move periodontal care away from the current one-size-fits all approach. Further, antibiotic use can be optimized. By predicting treatment outcomes for different antibiotic regimens, the model could help clinicians choose the most effective antibiotic dosage while minimizing unnecessary use. This is particularly important given the growing concerns about antibiotic resistance. By selecting the most appropriate treatment for each patient, this approach could potentially increase the success rate of periodontal treatments and reduce the number of patients experiencing disease progression despite treatment. Another possible outcome of implementing this machine learning model could help identify patients at higher risk of treatment failure, allowing for more intensive monitoring or alternative treatment strategies for these individuals [90].

However, it is important to note that while this approach shows promise, the authors emphasize the need for further validation, particularly prospective studies, before it can be implemented in clinical practice. Successful implementation would also require addressing challenges such as data privacy, clinician training, and integration with existing healthcare systems [91].

### 6.3. Transformer-Based Natural-Language Processing (NLP) and Deep Learning Radiology Pipelines

Transformer-based natural language processing (NLP) models can read and analyze the text notes contained in periodontal clinical charts. As an example, they can extract stage and grade determinations directly from these charts; fine-tuning bidirectional encoder representation for transformers (BERT) on 309 de-identified notes yielded 77% stage and 75% grade accuracy in a study performed in Canada, this model outperformed feature-engineered baselines [92]. This study provides some insight into how the BERT model’s performance compares to human clinicians in classifying periodontitis stages and grades. These researchers had controversially concluded that the BERT model’s performance is, in some cases, better than that of periodontal specialists. In reference to new, unseen data, the model achieved accuracy of 72% for grading and 66% for staging. These results are similar to the performance levels of periodontal specialists reported in a study by Oh et al., which found accuracies of 71.33% for staging and 64% for grading among clinicians with periodontal backgrounds [93]. The model significantly outperformed non-periodontal practitioners, the study suggests that the BERT model’s performance aligns more closely with specialist performance and exceeds that of non-specialists in classifying the stage and grade of periodontists. These comparisons are notable though the authors clarified the intention of the models are not to replace clinical judgment. These models are designed to serve as valuable support tools, particularly for general practitioners or those with limited experience in diagnosing periodontitis [94].

Based on a 2023 systematic review performed by Pethani & Dunn, several specific aspects of reporting were found to be lacking in dental NLP studies. The authors used a set of quality indicators to assess the completeness of reporting across the included studies [95]. The main areas where reporting were found to be insufficient include patient demographics, pre-processing information, data summary statistics, NLP methods descriptions, evaluation approach, code and data availability, ethical review and funding disclosures, and participant cohort selection [96]. In the review it was found that most studies did not provide detailed information about the demographic characteristics of the patients whose electronic dental records (EDR) were used to develop the NLP methods. Several studies did not provide complete information about the pre-processing steps used in their NLP pipeline; many studies failed to provide comprehensive data summary statistics, such as vocabulary size and document length; moreover, some studies did not fully describe the NLP methods used, including libraries, environment, and approach. The evaluation approach, including performance measures and methods of resampling and external validation, was not consistently or fully reported across all studies [97]. A consistent issue was code and data availability whereby none of the studies shared their code or deidentifies datasets, which limits reproducibility and further research, similarly, not all studies reported on their ethics review process or included funding disclosures. Participant cohort selection across papers was not transparent, a few studies did not fully describe how the study cohort was selected, including inclusion and exclusion criteria. The authors note that these gaps in reporting make it difficult to fully understand the NLP methods used, limit the ability to replicate studies, and hinder the synthesis of evidence across different studies in the field of dental NLP research [87].

Image-centric pipelines have matured similarly. Convolutional and vision-transformer networks have now achieved Dice coefficients (a statistical measure of similarity between two datasets) near 1 for tooth and bone segmentation and a high accuracy for automated staging on panoramic and periapical radiographs [98]. Integrated saliency maps consistently highlight the cementoenamel junction and crestal cortex, providing the visual transparency demanded by clinicians [97]. An independent ACM Digital Health study performed in China, confirmed these findings, and demonstrated that risk factor fusion (age, smoking, HbA1C) improves the robustness across the varying imaging devices [82]. These researchers used artificial intelligence, specifically a convolutional neural network (CNN) model, for diagnosing periodontitis in individuals. In producing a two-phase CNN model, the first phase aimed to preliminarily screen a pre-trained CNN that extracts features from dental images (panoramic radiographs), the model then outputs the screening predictive score for each panoramic radiograph [99]. In the next phase, the model produces a final diagnosis using periapical radiographs (each participant in the study had 10 different panoramic radiographs taken from various intraoral locations). Phase two uses a deep neural network (DNN) to classify full mouth apical radiographs; following a predictive scoring system, the model used the scores as features input into a Support Vector Machine (SVM) algorithm. The SVM produces a single final predictive score for each participant. By combining these two phases, the model provides both a preliminary screening based on panoramic radiographs and a more detailed, final diagnosis using multiple periapical radiographs. The model’s robustness was assessed under various confounding actors, displaying consistent accuracy across different scenarios. This approach allows for a comprehensive assessment of periodontitis, potentially improving diagnostic accuracy by leveraging distinct types of dental imaging [100]. Traditional diagnostic methods for periodontitis have limitations, including subjectivity and the need for high professional skills. Representative AI/ML models applied in periodontal and HIV contexts, their performance, and limitations are presented in Table 4.

### 6.4. Explainability and Validation in AI/ML for Periodontal Disease and HIV-1

In artificial intelligence, machine learning (ML) is the process of using a computer to identify and comprehend patterns in a large amount of data to create models for categorization and prediction using the training set. ML is separated into reinforcement learning, supervised learning, and unsupervised learning [102]. Recent advances highlight the importance of transparency and rigorous validation when applying AI/ML in dentistry and periodontology, particularly for populations affected by HIV. A 2023 analysis performed through the University of Oxford combined gradient-SHapley attribution with counterfactual testing, revealing calibration drift that disproportionately affected under-represented minority groups and proposing a governance checklist applicable to dental registries. Beall et al., investigated the use of a Deep Leaning (DL) approach to predict future disease diagnosis from Electronic Health Records (EHRs) for Population Health Management [103]. The explainable nature of the model (using SHapley Additive exPlanations (SHAP) values) could provide clinicians with insight into the factors contributing to a patient’s risk, aiding in clinical decision-making. The insights gained from this approach could inform health policy decisions, helping policymakers to focus on the most impactful preventive measures [104]. Another study, comparably—a systematic review of dental NLP—reported fewer than 10% of studies provided external validation or subgroup analysis, underscoring the need for transparent model cards before clinical use. Prospective multi-ethnic validation cohorts, integration of HIV-specific immunological markers and harmonized reporting standards remain priorities before widespread char-side adoption [105].

#### AI/ML Ethics and Governance: Clinical and Professional Implications

The responsible integration of artificial intelligence and machine-learning technologies into clinical dentistry requires careful attention to ethical, legal, and professional dimensions. Ensuring patient privacy and data protection is paramount, particularly when large, annotated image or biomarker datasets are shared across institutions. Compliance with established frameworks such as the Health Insurance Portability and Accountability Act (HIPAA) in the United States and the General Data Protection Regulation (GDPR) in Europe is essential, as emphasized in recent World Health Organization (2023) and American Dental Association (2023) position statements [106,107]. Equally important is the mitigation of algorithmic bias and inequity that can arise from unbalanced training datasets; the use of explainable-AI (XAI) approaches including SHAP, LIME, and Grad-CAM helps improve transparency and clinician trust [108,109]. Sustainable adoption of AI-driven diagnostics further depends on AI literacy and professional training, with curricula and continuing education programs designed to equip dental practitioners to critically evaluate and validate algorithmic outputs [110,111]. Finally, regulatory and institutional governance models should guide AI deployment through multidisciplinary ethics boards, performance audits, and transparent validation frameworks that align with current ethical and legal standards [112,113]. Addressing these challenges will allow AI-based diagnostic tools to complement, rather than replace, clinician expertise while ensuring equitable and ethically grounded application in routine oral healthcare.

### 6.5. How Does Artificial Intelligence Help in Academic Clinical Setting?

Evaluating periodontitis poses a diagnostic difficulty since the disease process involves intricate interactions between predisposing variables that are challenging for scientists and clinicians to completely understand. Due to these intricate aspects, the research of this illness benefits from the application of AI to better understand how these factors impact the etiology or diagnosis. Classification of periodontal disease can be facilitated using radiographically determined bone loss, risk factor codes, and periodontal data [94].

Artificial intelligence is already being implemented into electronic health record systems and pre- and postgraduate curricula. Mentioned in the study performed by researchers at the University of Oxford, these researchers used an artificial intelligence algorithm (Word2Vec) to create embedding from structured vocabulary commonly used in EHRs, then fed these embedding to a Bidirectional Gated Recurrent Unit (GRU) model to predict the likelihood of patients developing specific diseases [84]. Radiographic decision-support plug-ins deliver instant bone-loss quantification and recommended staging and codes during routine charting, reducing inter-examiner variability and save an estimated 2.5 min, or 180 s, per panoramic image [10]. NLP-based audit dashboards surface undocumented risk factors, prompting evidence-based template updates and closing undocumented risk factors, prompting evidence-based template updates and closing documentation gaps in student clinics [86]. Platforms such as IntelliGenes and Holomics accelerated hypothesis generation by allowing non-programmers to perform the feature selection amongst the differing omics types, in hours rather than weeks, thus shortening the cycle from biomarker discovery to animal or organoid validation.

### 6.6. Improving Periodontal Disease Diagnosis and Management with AI

AI is currently being used to help diagnose illnesses, estimate the prognosis of conditions, and create therapy regimens tailored to each patient. Also, to help dentists make clinical judgments in a timely manner, minimizing human mistakes, and delivering consistent quality care while lessening provider stress. Algorithms and AI-enhanced software can assist dentists in better communicating with patients and proving that treatment is necessary. The algorithms can attain accuracy comparable to that of a single doctor because they are based on millions of data points that have been learned through inputs [114].

According to research, one AI model exhibited periodontal diagnosing accuracy rates of 81% and 76.7% in premolars and molars, respectively, while another model has been used to recognize periodontitis by examining a patient’s subgingival plaque to differentiate microbial profiles. In a study conducted on premolars and molars, respectively, one AI model demonstrated periodontal diagnosis accuracy rates of 81% and 76.7% [83,88,89]. Table 5 summarizes recent studies applying AI to periodontal disease diagnosis and prediction, highlighting their accuracy and clinical potential. Another model has been utilized to identify periodontitis by analyzing a patient’s subgingival plaque to distinguish between distinct microbial profiles. Danks et al. analyzed periapical radiographs using a deep neural network to quantify periodontal bone loss [94]. Nakano and colleagues. demonstrated that deep learning has a 97% prediction accuracy in identifying oral malodor from bacteria [94]. The system’s overall proportion of critical points that were correct was 89.9%. Similarly, Tonetti et al. utilized panoramic pictures and a deep learning algorithm to identify and quantify periodontal bone loss, which was subsequently utilized to stage periodontitis [115]. The use of AI for classifying periodontal disease into clinical categories is summarized in Table 6.

## 7. Conclusions

This review synthesizes current evidence on the biological, clinical, and technological intersections between periodontal disease (PD) and HIV-1, highlighting how immune dysregulation, microbial shifts, and ART-related effects may modify periodontal outcomes in people living with HIV (PLWH). Across studies, PLWH exhibit altered inflammatory profiles, increased prevalence of dysbiotic microbial communities, and variable periodontal responses depending on immune status and ART history. Although emerging molecular and omics-based biomarkers show promise for detecting disease activity, their clinical utility remains limited by heterogeneous study designs and small sample sizes.

AI/ML applications in periodontology including imaging analytics, biomarker integration, and natural-language processing provide potential tools for improving PD risk prediction in PLWH. However, current models often rely on single-center datasets, lack external validation, and show vulnerability to domain shift. Ethical considerations, such as data governance, explainability, and protection of vulnerable populations, further highlight the need for careful implementation.

Overall, evidence supports a multifactorial interaction between HIV-1 and PD, but gaps in longitudinal data, standardized diagnostic criteria, and mechanistic studies limit definitive conclusions. Future research should prioritize rigorous multi-site cohorts, integration of clinical and omics datasets, and robust validation of AI-assisted tools. These efforts will be essential for developing reliable, equitable, and clinically actionable strategies to improve periodontal health in PLWH.

## Figures and Tables

**Table 1:** Modifiable and non-modifiable risk factors for periodontitis.

Individual Risk Factors		Local Risk Factors
Modifiable Risk Factors	Non-Modifiable Risk Factors	
Smoking	Age	Root proximity
Diabetes	Genetics	Enamel pearls and cemento-enamel projections
Obesity	Gender	Tooth malposition
Others such as stress, osteoporosis, alcohol consumption, nutritional deficiencies	Ethnicity	Root abnormalities such as palate-radicular grooves, cemental tears Uncommon factors such as sub-gingival restorations and open contacts

**Table 2:** Risk factors associated with HIV-1.

Non-Modifiable Risk Factors	Modifiable Risk Factors
Race	Sexual behavior
Ethnicity	Needle sharing
Economic and gender disparities	Untreated HIV-1 infection, metabolic syndrome, hypertension and obesity

**Table 3:** FDA-approved HIV-I medications.

Class of HIV-I Medications	Mode of Action	Refs.
Nucleoside reverse transcriptase inhibitors	NRTIs compete with natural deoxynucleotides for incorporation into a growing viral DNA chain. These NTRI’s include Abacavir, lamivudine emtricitabine, zidovudine and tenofovir disoproxil fumarate.	[39]
Non- Nucleoside reverse transcriptase inhibitors	These inhibitors act by binding to reverse transcriptase (RT) directly, NNRTIs inhibit this enzyme. Despite not being integrated into the viral DNA, NNRTIs prevent RT protein domains from moving, which is necessary for completing DNA synthesis. These include Efavirenz, etravirine, nevirapine, rilpivirine.	[37]
Protease Inhibitors	These bind to HIV-1 protease and block proteolytic cleavage of protein precursors necessary for producing viral particles. These include Atazanavir, darunavir, fosamprenavir, ritonavir, saquinavir, and tipranavir.	[40]
Fusion Inhibitors	Fusion inhibitors disrupt binding, fusion, and entry of HIV-1 virions into a human cell. Enfuvirtide binds to gp41 and disrupts membrane attachment.	[41]
Chemokine receptor 5 (CCR5) Antagonist	Maraviroc, a CCR5 antagonist, blocks the CCR5 receptor present on the T-cell to prevent viral attachment.	[42]
Integrase Inhibitors	Integrase inhibitors prevent the viral genome from inserting itself into the DNA of a host cell by blocking the action of integrase.	[43]
Post-attachment Inhibitors	These are monoclonal antibodies which bind to CD4 and inhibit viral entry into the cell. Ex: Ibalizumab	[44]
Pharmacokinetic Enhancers	These enhancers increase the plasma concentration of other HIV-1 drugs by inhibiting human CYP3A protein. HIV -1 patients are given the following regimens which are recommended by medical associations. These include: Tenofovir alafenamide, emtricitabine, and Bictegravir. Dolutegravir (emtricitabine or lamivudine).	[4,39,45]

**Table 4:** AI/ML models and methods for PD and HIV.

Method	Input Data	Model Type	Performance Metrics	Strengths	Limitations	Relevance to HIV/PD	Refs.
**Transformer-based NLP (BERT)**	Periodontal clinical notes (EHR/EDR)	Fine-tuned transformer (BERT)	Stage accuracy 77%; Grade accuracy 75%; Unseen data: 72% grading, 66% staging	Comparable to periodontal specialists; outperforms non-specialists	Requires large annotated datasets; limited external validation; poor demographic reporting	Could extract HIV-specific oral health details from EHRs; scalable for general practice	[92,93,94]
**Dental NLP** **Systematic Review**	Published NLP studies in dentistry	Quality assessment indicators	<10% external validation reported	Identifies reporting gaps; guides best practices	Lack of transparency; no code/data sharing; poor reproducibility	Highlights risk of bias in HIV/PD populations if reporting gaps persist	[87,95,97,100]
**CNN/Vision Transformers**	Radiographs (panoramic, periapical)	CNNs, Vision Transformers	Dice coefficients near 1 for tooth/bone segmentation; high staging accuracy	High accuracy; explainable with saliency maps	Needs diverse imaging datasets; device variability	Detects bone loss relevant to HIV-associated PD	[82,97,98]
**Two-Phase CNN + DNN + SVM Pipeline**	Panoramic + periapical radiographs	CNN (screening) → DNN (feature extraction) → SVM (final score)	Robust classification across imaging conditions	Multistage improves reliability; integrates risk factors (age, smoking, HbA1C)	Computationally intensive; requires multiple imaging modalities	Useful for HIV patients with systemic risk factors; enhances PD diagnosis accuracy	[99,101]

**Table 5:** Studies showing utilization of AI in periodontal disease diagnosis.

Sl. No.	Study	Findings	Ref.
1	Deep CNN based computer-assisted detection system in the diagnosis and prediction of PCT	PCT was diagnosed with an accuracy of 76.7% for molars and 81% for premolars.	[91]
2	Differentiating between periodontitis and healthy dental plaque	Accuracy of 78.8%	[87]
3	Detection of periodontitis for people with limited access to dental personnel and facilities in any healthcare setting	91.6% accuracy. Useful for people with limited access to dental clinics.	[116]

**Table 6:** Studies show the use of AI in the classification of periodontal disease.

Sl. No.	Study	Findings	Ref.
1	Classification of patients into generalized chronic periodontitis generalized aggressive periodontitis, and periodontal health by machine learning using 40 bacterial species	A support vector classifier using 40 bacterial species was useful to differentiate between PH, Ch P, and AgP. The relative bacterial load could distinguish between AgP and ChP.	[117]
2	A machine learning classifier trained with annotations from dentists gives pixel-wise inflammation segmentations of color-augmented intraoral photos	The classifier differentiates successfully between healthy and inflamed gingiva. The early diagnosis of periodontal diseases given by the classifier using photos acquired by intra-oral imaging devices can be advantageous for dentists and patients.	[118]
3	Classification of periodontal diseases using artificial neural network, support vector machine, and decision tree.	The decision tree and support vector system showed better accuracy in the classification of periodontal diseases.	[119]

## Data Availability

The original contributions presented in this study are included in the article. Further inquiries can be directed to the corresponding author.
